# Bacterial topography of the respiratory tract, including pulmonary site-of-disease, in people with active tuberculosis: a case-control study

**DOI:** 10.21203/rs.3.rs-9956587/v1

**Published:** 2026-06-09

**Authors:** Tinaye L. Chiyaka, Suventha Moodley, Donald Simon, Jane A. Shaw, Stephanus T. Malherbe, Yonghua Li, Robin M. Warren, Jose C. Clemente, Leopoldo N. Segal, Novel N. Chegou, Grant Theron, Charissa C. Marsh

**Affiliations:** DSI-NRF Centre of Excellence for Biomedical Tuberculosis Research, and SAMRC Centre for Tuberculosis Research, Division of Molecular Biology and Human Genetics, Faculty of Medicine and Health Sciences, Stellenbosch University, Tygerberg, Cape Town 7505, South Africa.; DSI-NRF Centre of Excellence for Biomedical Tuberculosis Research, and SAMRC Centre for Tuberculosis Research, Division of Molecular Biology and Human Genetics, Faculty of Medicine and Health Sciences, Stellenbosch University, Tygerberg, Cape Town 7505, South Africa.; Division of Immunology, Faculty of Medicine and Health Sciences, Stellenbosch University, Tygerberg, Cape Town 7505, South Africa.; Division of Immunology, Faculty of Medicine and Health Sciences, Stellenbosch University, Tygerberg, Cape Town 7505, South Africa.; Division of Immunology, Faculty of Medicine and Health Sciences, Stellenbosch University, Tygerberg, Cape Town 7505, South Africa.; Division of Pulmonary, Critical Care, and Sleep Medicine, New York University School of Medicine, New York, NY 10016, USA; DSI-NRF Centre of Excellence for Biomedical Tuberculosis Research, and SAMRC Centre for Tuberculosis Research, Division of Molecular Biology and Human Genetics, Faculty of Medicine and Health Sciences, Stellenbosch University, Tygerberg, Cape Town 7505, South Africa.; Department of Genetics and Genomic Sciences, Icahn School of Medicine at Mount Sinai, New York, NY 10029, USA; Division of Pulmonary, Critical Care, and Sleep Medicine, New York University School of Medicine, New York, NY 10016, USA; Division of Immunology, Faculty of Medicine and Health Sciences, Stellenbosch University, Tygerberg, Cape Town 7505, South Africa.; DSI-NRF Centre of Excellence for Biomedical Tuberculosis Research, and SAMRC Centre for Tuberculosis Research, Division of Molecular Biology and Human Genetics, Faculty of Medicine and Health Sciences, Stellenbosch University, Tygerberg, Cape Town 7505, South Africa; DSI-NRF Centre of Excellence for Biomedical Tuberculosis Research, and SAMRC Centre for Tuberculosis Research, Division of Molecular Biology and Human Genetics, Faculty of Medicine and Health Sciences, Stellenbosch University, Tygerberg, Cape Town 7505, South Africa

**Keywords:** tuberculosis, respiratory tract, microbiota, site-of-disease

## Abstract

**Background:**

No comprehensive characterization of the respiratory tract (RT) microbiota has been done in people with tuberculosis (TB), a leading global cause of death.

**Methods:**

16S rRNA gene sequencing was done on upper RT (URT; oral-washes, naso- and oro-pharyngeal swabs, supraglottic fluid), sputum and lower RT [LRT; bronchoalveolar lavage fluid (BALF) and protected specimen brushings] specimens from HIV-negative people with Xpert MTB/RIF-confirmed TB (cases; n=17) and healthy controls (n=11). In addition to their diseased lobe, cases had their non-diseased lobe sampled.

**Results:**

The LRT had the lowest α-diversity and β-diversity differed compared to other respiratory compartments. In cases, *Mycobacterium*relative abundance was highest in the diseased lobe 1.537% (CI 0–3.114), followed by the nasopharynx 0.059% (0.012–0.105), non-diseased lobe 0.054% (0–1.620), oropharynx 0.003% (0–0.010) and sputum 0.002% (0–0.004). Compared to the URT and sputum, cases’ LRTs were *Mycobacterium-* and *Moraxella*-enriched (*Erythromicrobium*-enriched versus sputum only). In paired comparisons of diseased versus non-diseased lobes in cases, the only differential taxon was *Mycobacterium*. Amongst non-diseased lobes, those of cases versus controls had reduced α-diversity with *Mycoplasma*-enrichment and *Moraxella-* and *Klebsiella-*depletion.

**Conclusion:**

Compared to healthy people, those with TB have a less diverse LRT microbiota, characterized by *Mycobacterium*-enrichment (within the diseased lobe and surprisingly least so in sputum) and depletion of taxa associated with healthy people. In people with TB, most microbial DNA is not mycobacterial within the diseased lobe and even the non-diseased lobes of cases are microbially distinct from controls. These findings provide a foundation for understanding respiratory tract host-microbiome interactions in TB.

## Background

Tuberculosis (TB) is a public health catastrophe, particularly in low- and middle-income countries. In 2024, the World Health Organization (WHO) estimated ~ 10.7 million incident TB cases and 1.23 million deaths as a result of TB^1^. The human microbiota is a critical mediator of human health^2–5^ but inadequately studied in TB^6–8^, particularly in the lungs which are the primary site-of-disease. These knowledge gaps preclude the design and evaluation of interventions that could target the microbiota to avert poor clinical outcomes associated with TB.

The respiratory tract is a continuous ecosystem with a gradient of conditions (e.g., pH) and microbial biomass^9^. It is divided into the upper respiratory tract (URT), which spans from the anterior nares, nasal cavity, paranasal sinuses, pharynx and supraglottic portion of larynx, and lower respiratory tract (LRT) composed of the trachea and lungs^9^. Few studies have described the respiratory microbiota in TB with most focussing only on sputum which, although routinely collected for TB diagnosis, is a mix of URT and LRT microbes^10–12^.

Bronchoscopy with bronchoalveolar lavage (BAL), although invasive and expensive, remains the gold standard for sampling the LRT including the site-of-disease in pulmonary TB^13,14^. Some studies have examined BAL fluid (BALF) microbiota from people with TB^15–17^, describing BALF associated microbiota in TB. However, in these studies, measures to minimise contamination of low biomass BALF, such as bronchoscope washes and limited suctioning (to reduce biomass disturbance), are not typically done. Furthermore, no studies have sampled anatomical sites across the full respiratory tract within the same individuals with TB. To date, two studies^18,19^, have compared paired lung lobes with and without disease involvement in people with TB, however, participants in one study had received two weeks of TB treatment prior to bronchoscopy, potentially altering microbiota profiles.

To address these limitations and knowledge gaps, we comprehensively characterized in healthy controls and people with TB the full respiratory tract microbiota across multiple sites, beginning in the oral cavity, followed by the oropharynx, nasopharynx, supraglottis, sputum, non-diseased lung lobe (in healthy controls and people with TB), and diseased lung lobes (in people with TB).

## Methods

### Ethics

The study was conducted in accordance with the Declaration of Helsinki and approved by the Stellenbosch University Health Research Ethics Committee (N16/05/070). Informed consent was obtained from all participants.

### Participants and definitions

People (≥ 18 years old) were sourced from a diagnostic evaluation study in Cape Town, South Africa that consecutively recruited people with presumptive pulmonary TB and healthy controls (ClinicalTrials.gov NCT03350048). TB cases were classified based on a positive sputum Xpert MTB/RIF result. Controls were from the same setting without clinical, radiological or mycobacterial evidence of TB. Cases underwent chest X-ray to identify which lobe was non-diseased and diseased (cases were pre-treatment). For controls, any lobe with no contraindications visible on chest X-ray was chosen for bronchoscopy.

### Specimen collection and classification

Pre-bronchoscopy, swabs (Zymo Research, Irvine, USA) from the tonsils and posterior oropharyngeal mucosa (oro-swab), and nasopharyngeal mucosa (naso-swab) were collected and each stored in 2mL DNA/RNA Shield (Zymo Research) at −80°C. An oral wash, expectorated sputum, and induced sputum were collected^7^. Sterile water and saline were used as DNA sampling controls (background) for oral washes and induced sputum. Before bronchoscopy, saline was flushed through a sterile, single-use Yankauer and stored as background. The Yankauer (attached to a mucous trap) was advanced to the supraglottic space (posterior oropharynx) under direct visualisation with suctioning for a few seconds to collect supraglottic fluid. Before bronchoalveolar lavage (BAL), sterile saline was flushed through the bronchoscope and stored as background. BALF (both cases and controls) and protected specimen brushings (PSBs; cases only) were collected using a modified bronchoscopy procedure to minimise contamination (**Supplementary methods**). In cases, specimens were collected first in the non-diseased lobe followed by the diseased lobe. Specimens were categorised as URT (oral wash, oro-swab, naso-swab and supraglottic fluid), sputum (expectorated and induced) or LRT (BALF, PSBs).

### Specimen processing

Oral washes, sputa, and background specimens were decontaminated using N-acetyl-L-cysteine (NALC), pelleted (3,217×*g*) and resuspended in 2mL phosphate buffer (pH 6.8; BD, Johannesburg, South Africa). These aliquots and raw aliquots of supraglottic fluid, BALF and PSBs were stored at −80°C. Prior to DNA extraction, we did a lysozyme (30min, 37°C) incubation step and a freeze-thaw cycle (−20°C) to lyse cells. DNA was extracted using the QIAamp DNA Mini kit (QIAGEN, Hilden, Germany), according to manufacturer instructions on the QIAcube platform^20^.

### Sequencing and analysis

16S rRNA gene sequencing (V4 region, 150bp paired-end reads) was done using Illumina MiSeq^6,7^. Sequences were pre-processed using QIIME 2 2020.2^21^ and clustered into amplicon sequence variants (ASVs) using DADA2^22^. Taxonomy was assigned at 99% similarity against the GreenGenes reference database^23^. Potentially contaminating taxa were identified using *decontam* (v1.14.0)^24^ but not removed from analyses and rather flagged (in red) in analyses. α- and β-diversity metrics were calculated using *vegan*^25^. Non-parametric tests, including the Mann-Whitney U test and the Kruskal-Wallis test with Dunn’s post-hoc comparison, were used for α-diversity comparisons. Permutational Multivariate Analysis of Variance (PERMANOVA) was used for β-diversity. Differential abundance comparisons were done using *DESeq2*^26^ and multiple comparisons adjusted using the Benjamini-Hochberg procedure. Sequencing data are available on the Sequence Read Archive (SRA; PRJNA1367636).

## Results

### Study population

Of the 28 people, cases (n = 17) compared to controls were more likely to be underweight (n = 11) and have previous TB (Table 1).

### Respiratory specimens’ microbial profiles are distinct from background controls

α-Diversity was lower in URT, sputum, and LRT compared to each background control and β-diversity differed between background and URT, sputum and LRT specimens (**Figure S1B-C**). Potentially contaminating amplicon sequence variants (ASVs) identified are listed in **Supplementary Table S1**.

### Within-individual taxonomic differences in the lower respiratory tract compared to the upper respiratory tract and sputum

When specimens of the same type were compared by TB status, differences in α-diversity occurred (URT p<0.001, sputum p=0.002, LRT p=0.039; **Supplementary Table S2**). β-Diversity differed in URT and sputum (p=0.003 and 0.001) but did not in LRT (p=0.201).

#### Controls:

**Figure 1:**
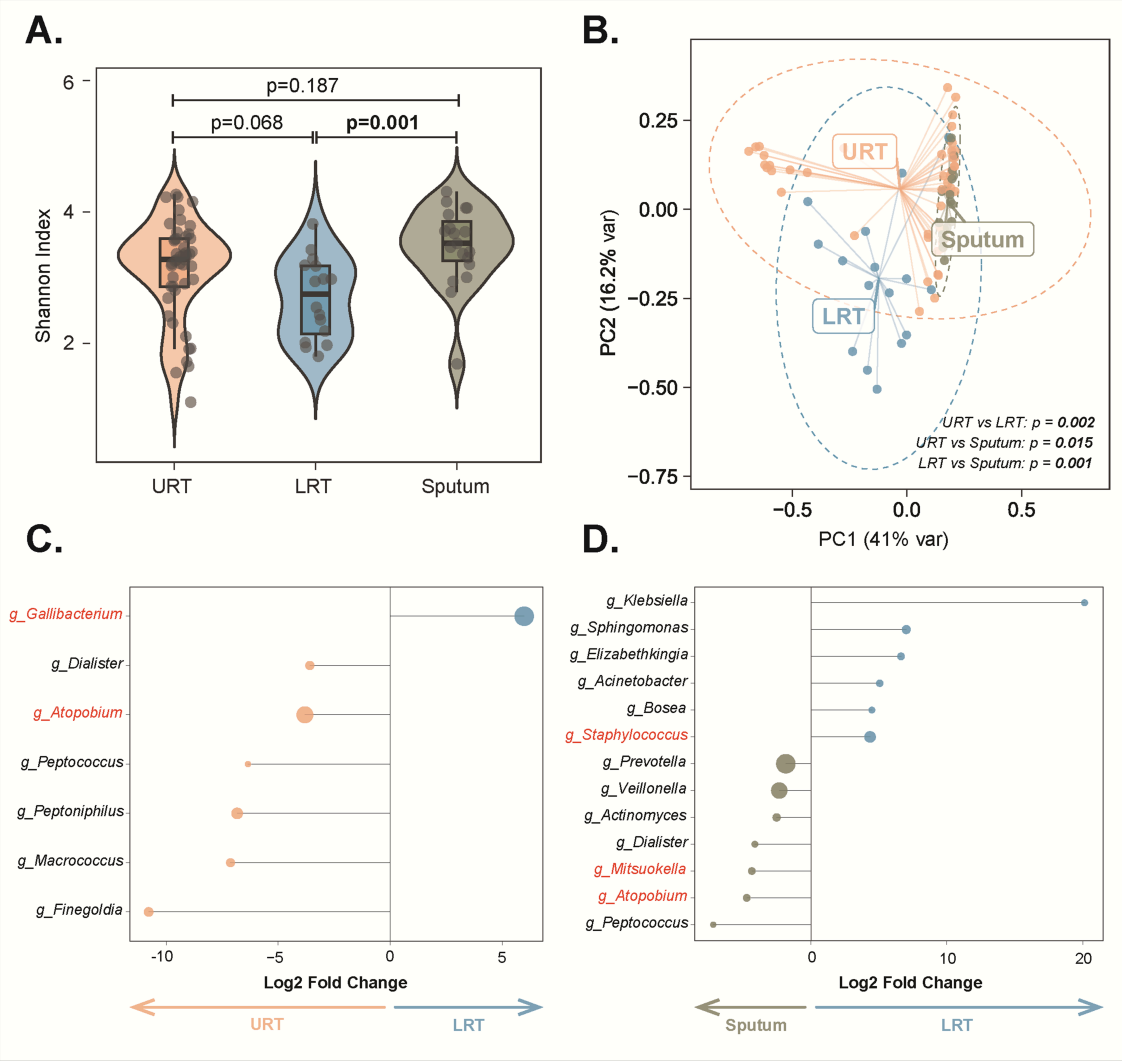
Diversity and composition of respiratory tract in controls: **A.** α-Diversity comparisons across respiratory compartments [Upper respiratory tract (URT), sputum and lower respiratory tract (LRT)]; **B.** β-Diversity comparisons showing compositional differences; Bubble plot showing differentially abundant taxa in **C.** URT versus LRT and **D.**Sputum versus LRT comparisons. Dot size corresponds to mean relative abundance of each taxon. The most differential taxa appear higher on y-axes (based on adjusted p-value). Red font indicates potentially contaminating taxa.

LRT α-diversity was lower than in sputum (p=0.001) not the URT (p=0.068). The URT and sputum were similar (p=0.187). β-Diversity differed between LRT versus URT and sputum (p=0.002 and p=0.001, respectively), and URT versus sputum (p=0.015). The LRT was *Dialister-, Peptococcus-* and *Peptoniphilus-*depleted versus the URT; *Klebsiella-, Sphingomonas-* and *Elizabethikingia-*enriched but *Prevotella-, Veillonella-* and *Actinomyces-*depleted versus sputum (Figure 1A-D).

#### Cases:

**Figure 2:**
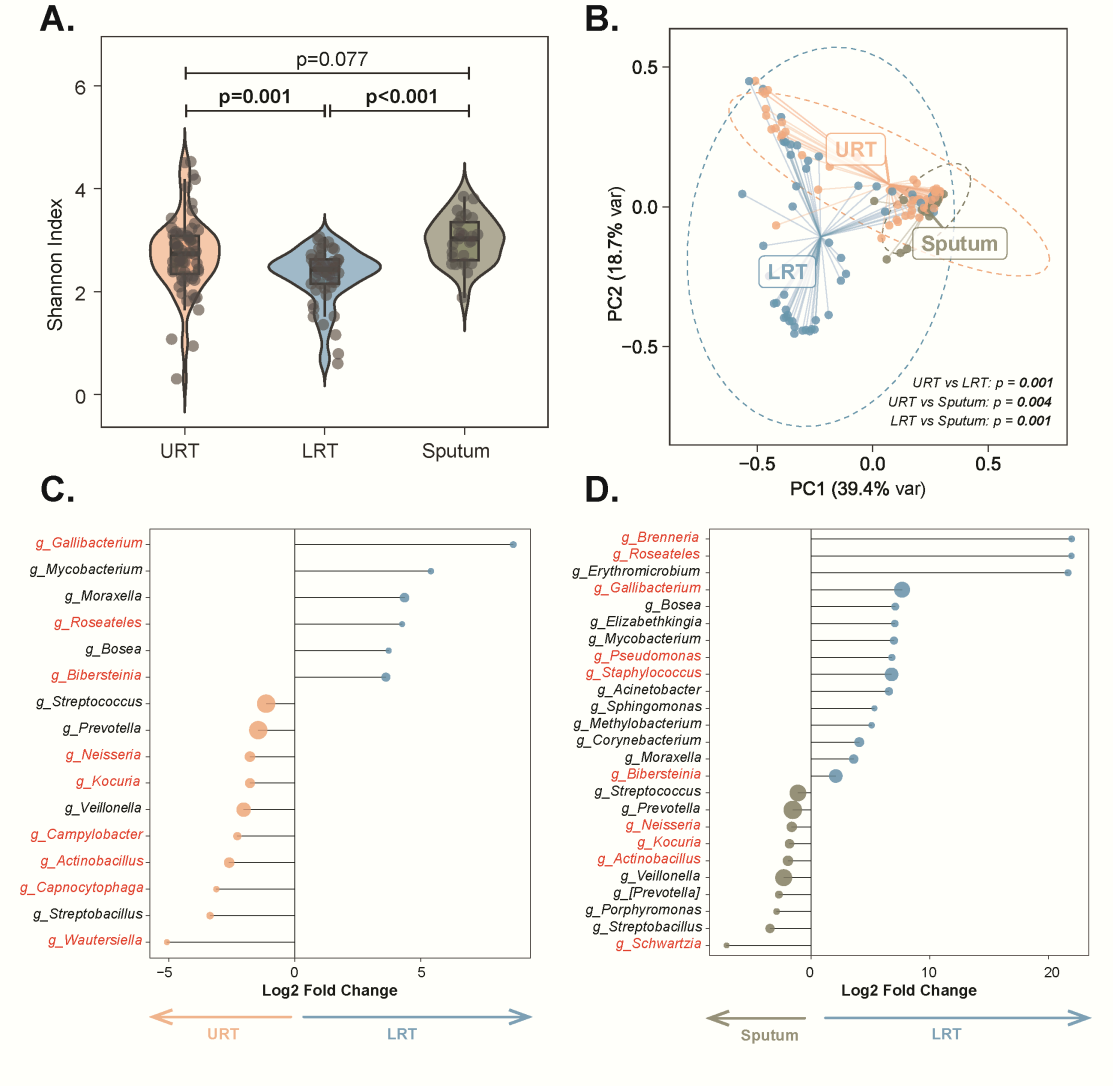
Cases’ have a distinct lower respiratory tract (LRT) microbiota: **A.** α-Diversity comparisons showing the LRT is the least diverse respiratory compartment versus the upper respiratory tract (URT) and sputum; **B.** β-Diversity comparison showing LRT, URT and sputum microbiota is distinct; Bubble plot showing differentially abundant taxa in **C.** URT versus LRT and **D.** Sputum versus LRT comparisons. Dot size corresponds to mean relative abundance of each taxon. The most differential taxa appear higher on y-axes (based on adjusted p-value). Red font indicates potentially contaminating taxa.

α-Diversity in the LRT was lower than the URT (p=0.001) and sputum (p<0.001) but did not differ between URT and sputum (p=0.077). β-Diversity differed between LRT versus URT and sputum (both p=0.001), and URT versus sputum (p=0.004). Compared to the URT, the LRT was *Mycobacterium-, Moraxella-,* and *Bosea*-enriched but *Streptococcus-*, *Prevotella-* and *Veillonella-*depleted. The LRT was *Erythromicrobium-, Bosea-* and *Mycobacterium*-enriched but *Streptococcus-*, *Prevotella-* and *Veillonella-*depleted versus sputum (Figure 2A-D).

### *Mycobacterial* load and relative abundance at different respiratory tract sites in cases

**Figure 3:**
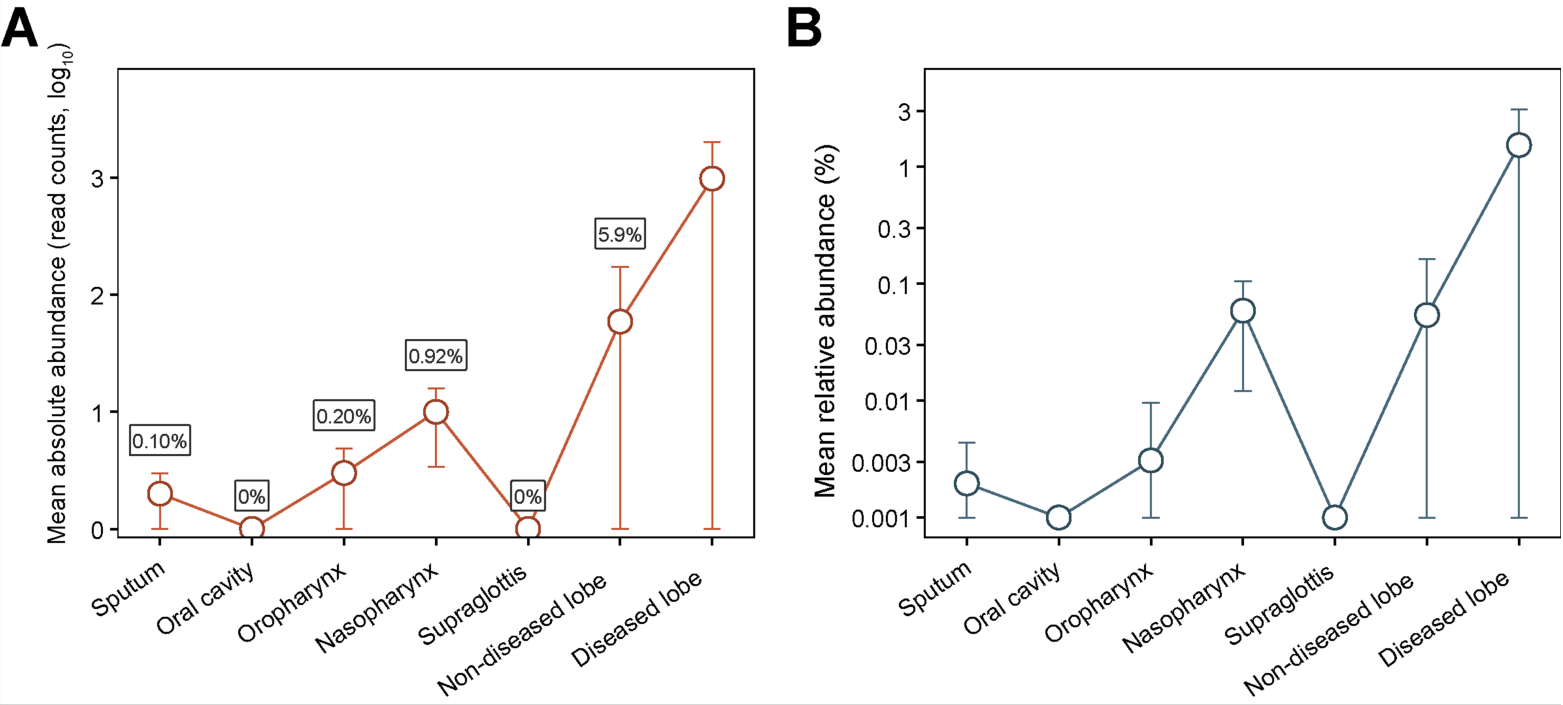
*Mycobacterium* is a small minority at the site-of-disease: **A.** *Mycobacterium* mean absolute read counts and proportion (%) relative to quantity in diseased lobe; **B.** Mean relative abundance of *Mycobacterium* across the respiratory tract. Error bars are 95% confidence interval (CI).

Cases’ diseased lobes had the highest absolute mean (95% confidence interval) *Mycobacterium* read count 981 (0–2023), followed by the non-diseased lobe 57 (0–173), nasopharynx 9 (2–15), oropharynx 1 (0–4), sputum 1 (0–2), with no *Mycobacterium* detected in the supraglottis or oral cavity. This translates into the sputum, oropharynx, nasopharynx, and non-diseased lobe having 0.10%, 0.20%, 0.92% and 5.9% respectively of the quantity of *Mycobacterium* at the diseased lobe. The mean proportion of total bacterial reads from each site that were *Mycobacterium* (relative abundance) were also highest in the diseased lobe 1.537% (95% confidence interval 0–3.114), followed by nasopharynx 0.059% (0.012 – 0.105), non-diseased lobe 0.054% (0 – 1.620); oropharynx 0.003% (0 – 0.010) and sputum 0.002% (0 – 0.004, Figure 3A-B).

### Diseased and non-diseased lobes of cases only differ by *Mycobacterium* abundance

**Figure 4:**
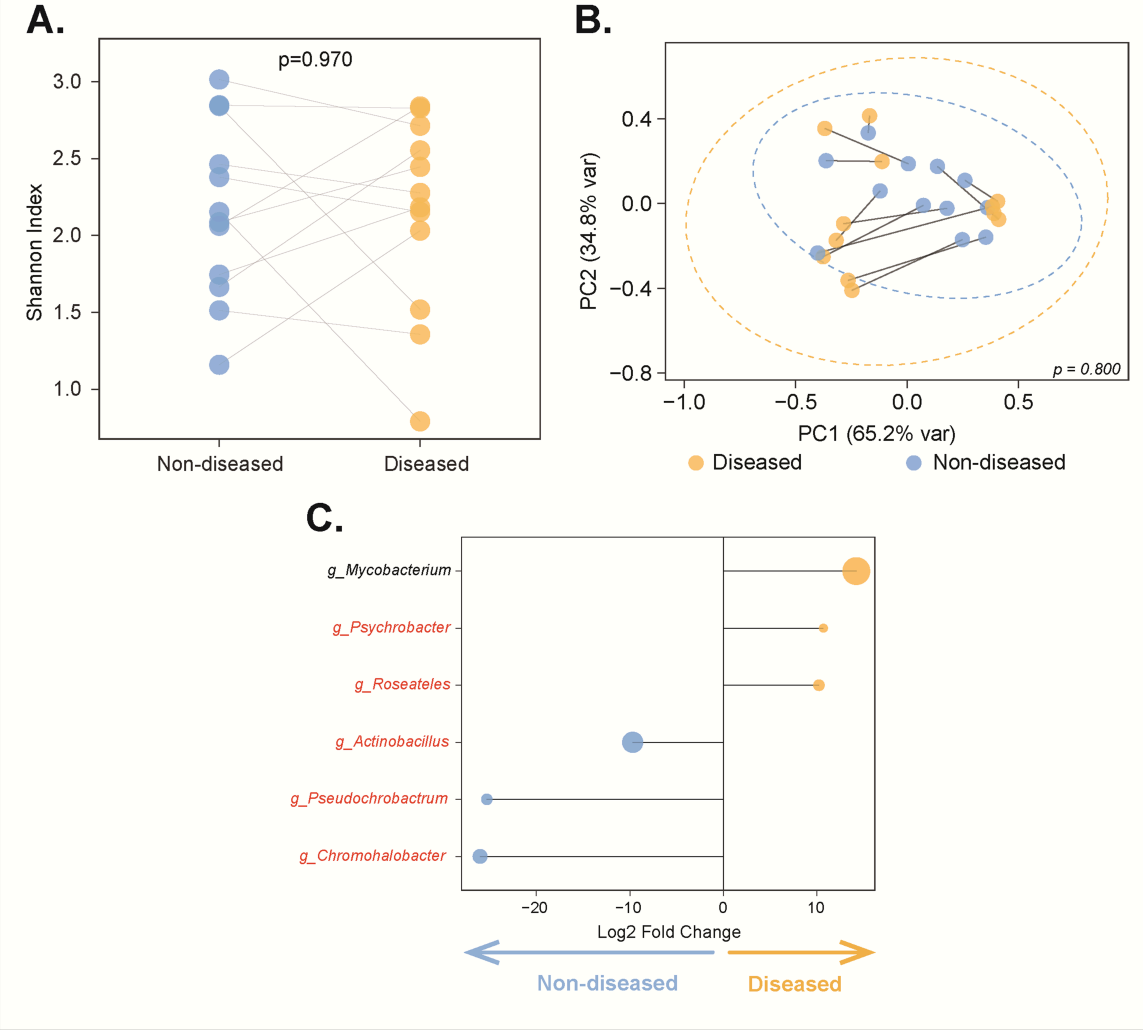
Bronchoalveolar lavage fluid (BALF) microbiota comparisons in diseased and non-diseased lobes within individuals: **A-B.** Paired α- and β-diversity comparisons of diseased and non-diseased lung lobes show no differences; **C.** Bubble plot showing *Mycobacterium* is the only differential taxa in diseased versus non-diseased lung lobes. Dot size corresponds to mean relative abundance of each taxon. The most differential taxa appear higher on y-axes (based on adjusted p-value). Red font indicates potentially contaminating taxa.

Within-individual diseased versus non-diseased lung lobe comparisons in cases showed α-diversity did not differ when either BALF or PSBs were compared (p=0.970, p=0.250). Similarly, β-diversity did not differ (p=0.800, p=0.923). In BALF, diseased lobes were *Mycobacterium*-enriched versus non-diseased lobes, and for PSBs, there were no differential taxa (Figure 4A-C, **Figure S3A-B**).

### People with tuberculosis and have reduced airway microbial diversity compared to controls

**Figure 5:**
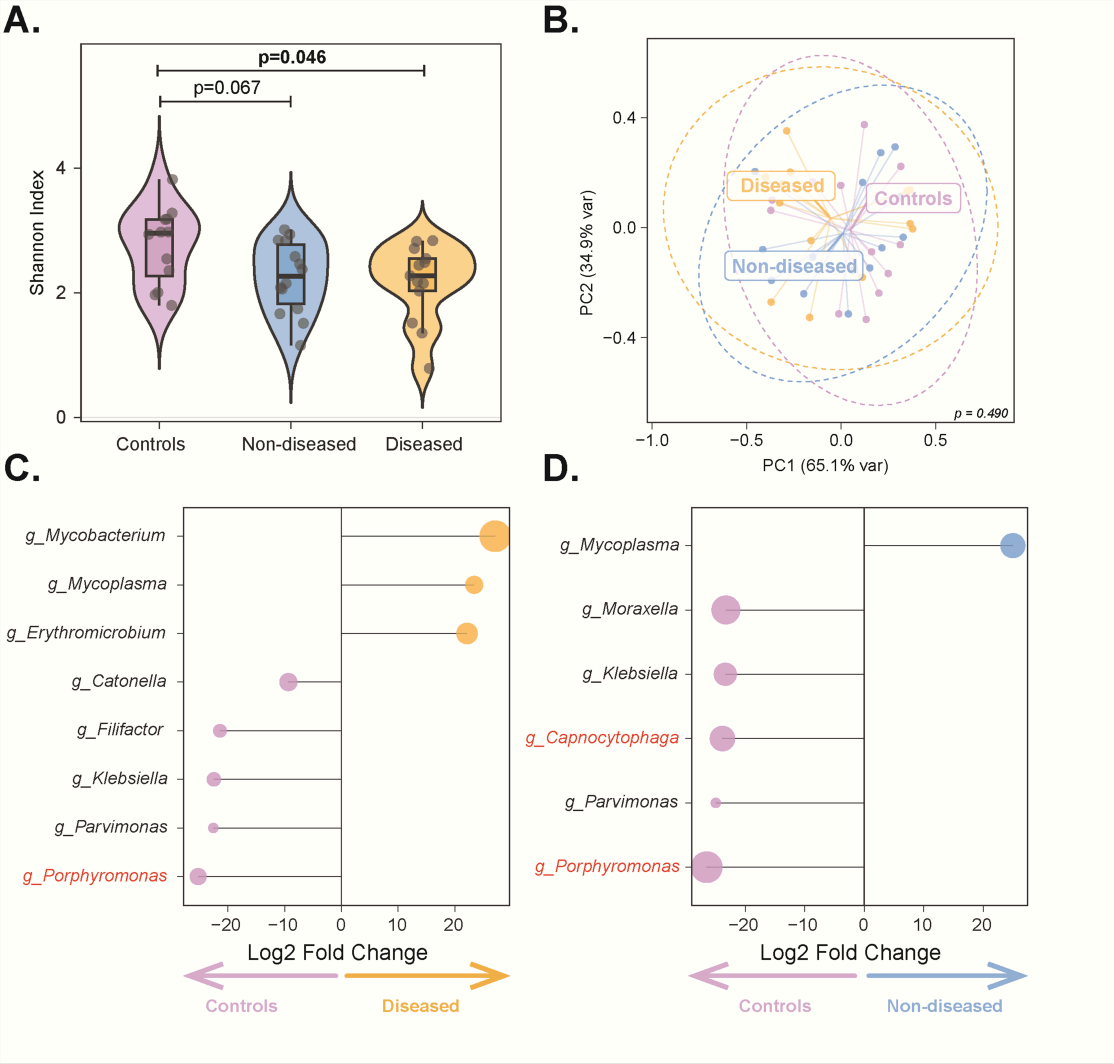
Bronchoalveolar lavage fluid (BALF) microbiota of cases (diseased and non-diseased lobes) versus controls: **A.** α-Diversity comparison showing both diseased and non-diseased lung lobes have reduced microbial diversity versus controls; **B.** β-Diversity comparison between cases and controls BALF; Bubble plot showing differentially abundant taxa in **C.** Diseased lobes versus controls and **D.** non-diseased lobe versus controls. Dot size corresponds to mean relative abundance of each taxon. The most differential taxa appear higher on y-axes (based on adjusted p-value). Red font indicates potentially contaminating taxa.

In BALF, cases had lower α-diversity versus controls (diseased and non-diseased lobes in cases each versus the non-diseased lobe in controls; p=0.046, p=0.067) and no β-diversity differences were observed (p=0.490). Cases’ diseased lobes were *Mycobacterium-, Mycoplasma-*, and *Erythromicrobium-*enriched, but *Catonella-, Filifactor-* and *Klebsiella*-depleted versus controls. Cases’ non-diseased lobes were *Mycoplasma-*enriched but *Moraxella-, Klebsiella-* and *Parvimonas-*depleted versus controls (Figure 5A-D).

## Discussion

Our data shows 1) site-of-disease (lungs) followed by nasopharynx, oropharynx and sputum respectively have the highest abundance of *Mycobacterium* in people with TB; 2) in people with TB, *Mycobacterium* DNA is a small minority of total microbial DNA at each site but most abundant at the site-of-disease and least so in sputum; 3) the LRT (in both cases and controls) is the least diverse compartment and microbially distinct from both URT and sputum; and 4) in cases the diseased and non-diseased lung lobes have similar diversity and composition, however, diseased lobes are *Mycobacterium*-enriched and overall cases have lower microbial diversity throughout the respiratory tract compared to controls. Together, these data comprehensively describe the respiratory microbial profile of people with TB.

The highest read count (absolute abundance) of *Mycobacterium* was in the lungs (diseased lobe then non-diseased lobe), followed by the nasopharynx, oropharynx, and sputum. The low *Mycobacterium* abundance in sputum is consistent with studies reporting difficult of detecting *Mycobacterium* in sputum using 16S rRNA gene sequencing^7,11,12^. Interestingly, nasopharyngeal swabs had, other than site-of-disease, has the highest *Mycobacterium* abundance. In a systematic review and meta-analysis of the diagnostic performance of different oral sample types for TB diagnosis, nasopharyngeal aspirates had the highest sensitivity point estimate and have shown promise for TB diagnosis in decedents^27,28^. Findings from our study provide the first view on the distribution of *Mycobacterium* throughout the respiratory tract but highlights the need for further research using sensitive approaches such as metagenomics, which may be less biased.

We show the LRT is less diverse and has a distinct microbial environment compared to the URT and sputum. In healthy people, the LRT is a low microbial biomass niche, maintained by efficient microbial clearance to preserve efficient gas exchange^29^ but can be disrupted during disease^30^. In cases the LRT was *Mycobacterium-*, *Moraxella-*, and *Bosea-*enriched versus the URT, indicating a shift towards dominance by specific taxa supported. Increased pulmonary abundance of *Moraxella* has been associated with chronic obstructive pulmonary disorder^7,31^ and is an opportunistic pathogen in immunocompromised individuals^32,33^. *Bosea* is enriched in the sputum of people with TB compared to those without TB^34^ and, as in our study *Bosea* was more enriched in the LRT than sputum, the LRT is likely a *Bosea* reservoir in people with TB.

We found comparable α- and β-diversity and *Mycobacterium-*enrichment in the diseased versus non-diseased lobes, consistent with a study from China^18^. We speculate that deeper sequencing approaches, like metagenomics, may reveal more differential taxa in diseased versus non-diseased lung lobes within people with TB. Studies examining the BALF microbiota reported lower α-diversity^17^ and *Mycobacterium*-enrichment in people with TB compared to symptomatic controls^15,16^. Consistent with these findings, we found lower α-diversity and *Mycobacterium*-enrichment in diseased lobes versus healthy controls. Even though the microbial diversity of the diseased versus non-diseased lobes of cases did not differ, we still observed *Mycobacterium* enrichment in diseased lobes. Longitudinal studies which sample the lung microbiota in the stages preceding TB disease are necessary to determine whether *Mycobacterium* shapes the diseased lung microbiota likely favouring colonisation with pathobionts.

Our study has strengths and limitations. A key strength is that we intensively sampled both the upper and lower airways (with appropriate background controls and a specialised bronchoscopy method) to provide a comprehensive description of the respiratory tract microbiota in pulmonary TB. We acknowledge that while the CXR has limited resolution compared to computed tomography for assessing disease involvement, but it can still be useful in discerning the relative diseased status of lobes. Bronchoscopy is inherently prone to microbial cross contamination, which is why used a modified bronchoscopy procedure designed to limit this as well using a bioinformatic approach with background controls to flag potentially contaminating taxa. Lastly, our study is cross-sectional and does not include people living with HIV, however, we provide important baseline data which can be used for justifying future longitudinal studies.

In summary, we provided the first comprehensive characterization of the microbiome throughout the respiratory tract, describing the relative abundance of different taxa in both people with TB and healthy people. This, together with the within-patient measurement of *Mycobacterium*, shows a landscape where the LRT has low microbial diversity relative to the URT and, with TB disease, additional potentially pathogenic taxa in the LRT.

## Supplementary Material

Supplementary Files

This is a list of supplementary files associated with this preprint. Click to download.

• ChiyakaetalSupplementaryData.docx

• ChiyakaetalSupplementaryTables.xlsx

## Figures and Tables

**Table 1:** Demographic and clinical characteristics. Data are median (IQR) or n (%).

Characteristics	Cases (n = 17)	Controls (n = 11)	*p-value*
**Sex**			
Females	6 (35)	8 (72)	0.053
Males	11 (65)	3 (28)	
**Age (years)**	43 (34–48)	34 (25–40)	0.159
**Ethnicity**			
Mixed ancestry	16 (94)	10 (91)	0.748
White	1 (6)	1 (9)	
**BMI (kg/m^2^)**			
Underweight (< 18.5)	8 (47)	1 (9)	**0.036**
Normal (18.5–24.9)	7 (41)	3 (27)	
Overweight (≥ 25.0)	2 (12)	7 (64)	
**Prior TB**			
Yes	9 (53)	0 (0)	**0.003**
No	8 (47)	11 (100)	
**Smoking**			
Never	3 (18)	3 (27)	0.544
Current	13 (76)	8 (73)	
Stopped < 6 months ago	1 (6)	0 (0)	
**Alcohol**			
No	8 (47)	5 (45)	0.934
Yes	9 (53)	6 (55)	
**Non-TB antibiotics**			
Yes	1 (6)	0 (0)	0.413
No	10 (59)	11 (100)	
Unknown	6(35)	-	-

## Data Availability

Sequencing data used in this study is available in the Sequence Read Archive (SRA; PRJNA1367636).
